# Association between knowledge and attitudes towards advance directives in emergency services

**DOI:** 10.1186/s12910-021-00646-y

**Published:** 2021-06-22

**Authors:** Silvia Poveda-Moral, Pilar José-Maria de la Casa, Pere Sánchez-Valero, Núria Pomares-Quintana, Mireia Vicente-García, Anna Falcó-Pegueroles

**Affiliations:** 1 School of Nursing, Faculty of Medicine and Health Sciences, University of Barcelona, Barcelona, Spain; 2 Escola Universitària D’Infermeria I Teràpia Ocupacional de Terrassa (EUIT), Universitat Autònoma de Barcelona, Calle de La Riba, 90, 08221 Terrassa, Barcelona, Catalonia Spain; 3 Fundación Sanitaria de Mollet, Mollet del Vallés, Spain; 4 Coordinador Territorial Servicio de Emergencias Médicas, Generalitat de Catalunya, Barcelona, Catalonia Spain; 5 School of Nursing. Faculty of Medicine and Health Sciences. Consolidated Research Group SGR 269 Quantitative Psychology, University of Barcelona, Barcelona, Spain

**Keywords:** Advance directives, Hospital emergency department, Emergency medical services, Attitudes, Knowledge

## Abstract

**Background:**

Implementing the routine consultation of patient advance directives in hospital emergency departments and emergency medical services has become essential, given that advance directives constitute the frame of reference for care personalisation and respect for patients’ values and preferences related to healthcare. The aim of this study was to assess the levels and relationship of knowledge and attitudes of nursing and medical professionals towards advance directives in hospital emergency departments and emergency medical services, and to determine the correlated and predictor variables of favourable attitudes towards advance directives.

**Methods:**

Observational, descriptive, and cross-sectional study. The study was conducted in the emergency department of a second-level hospital and in the emergency medical service. Data collection was performed from January 2019 to February 2020. The STROBE guidelines were followed for the preparation of the study.

**Results:**

A total of 173 healthcare professionals responded to the questionnaire. Among them, 91.3% considered that they were not sufficiently informed about advance directives, and 74% acknowledged not having incorporated them into their usual practice. Multinomial analysis indicated a statistically significant relationship between the variable emergency medical service and having more favourable attitudes towards consulting the advance directives in their practical application (OR 2.49 [95% CI 1.06–5.88]; *p* = 0.037) and compliance in complex scenarios (OR 3.65 [95% CI 1.58 − 8.41]; *p* = 0.002). Working the afternoon and night shift was a predictor variable for obtaining a higher score with respect to attitudes in complex scenarios.

**Conclusion:**

There is an association between the level of knowledge that nursing and medical professionals have about advance directives and the scores obtained on the attitude scales at the time of practical implementation and in complex scenarios. This shows that the more knowledge professionals have, the more likely they are to consult patients' advance directives and to respect their wishes and preferences for care and/or treatment.

## Background

It is known that scientific and technical advances in recent years have had a positive influence by increasing life expectancy. The improvement of global healthcare in our environment has produced a percentage increase in the demand for care from emergency services in recent decades [1]. A systematic review that assessed thirty-one studies indicated that the most frequent users of hospital emergency departments and emergency medical services were older adults, patients with complex chronic diseases, patients with psychiatric comorbidities, and patients with low socioeconomic levels [2]. According to data from the World Health Organisation, there will be two billion individuals aged 60 and over in 2050. This is a population with increasingly complex pathologies and comorbidities that will require urgent care to treat episodes of acute exacerbations [3]. This reality is having an impact on most health systems. Greater use of hospital emergency departments can cause saturation and collapse of services. Serving increasingly aging populations means that the assessments made by the professionals working at emergency services will be substantially more complex [4–6]. In many cases, chronic and multi-pathological pictures are accompanied by some type of cognitive impairment that makes it difficult for the patient to be involved in decision-making [2, 7]. Free choice and respect for patient autonomy are important principles in our health care systems. In fact, current evidence shows that comprehensive, person-centred care helps to reduce unwanted hospitalisations and increases patients' self-care and professionals' satisfaction [8, 9]. These benefits were already raised in 2013 by the American Geriatrics Society, together with several North American emergency societies (American College of Emergency Physicians, Emergency Nurses Association, and Society for Academic Emergency Medicine) [10].

In this context, implementing the routine consultation of patient advance directives (AD) in hospital emergency departments and emergency medical services becomes essential, given that ADs constitute the frame of reference for care personalisation and respect for patients’ values and preferences related to healthcare [11]. ADs allow decision-makers to set goals and preferences for future medical care and treatments, which should be respected and met in the absence of patients’ ability to express themselves [12].

Previous studies have indicated that it is difficult to know patients’ preferences in emergency services. According to these studies, ignorance about ADs, registration mechanisms, and the normative aspects that regulate them, as well as the lack of skills of professionals in managing them, constitute the main factors hindering the consultation and implementation of AD in these services [13, 14]. Likewise, in addition to posing a clear threat to respect for individuals’ autonomy of decision [15], these obstacles described in the literature can generate conflictive situations from an ethical and legal perspective [16]. They can also lead to a professional practice based on "defensive medicine, when professionals are afraid of receiving a complaint from patients or relatives [17], or therapeutic futility, when the professional is unable to recognize that life has its limits and that there are some procedures that violate the autonomy and dignity of the person.

In the context of Spanish law and regulations regarding ADs, the General Health Law 14/1986 on April 5, made explicit the need to regulate actions to enforce the right to health protection recognized in the Spanish Constitution. Articles 9 to 11 established the rights and duties of users of the National Health System. The new legal framework represented a commitment to a model more focused on the principle of autonomy of the person, abandoning the traditional model of care based on medical paternalism. Further, the Convention on Human Rights and Biomedicine (Oviedo Convention) [18], which came into force on January 1 2000, permitted a step forward in the recognition of living wills in establishing that ‘…the wishes previously expressed regarding a medical intervention by a patient who, at the time of the intervention, is not able to express himself, will be taken into consideration’. The legislation on advance directives in Spain and its autonomous communities is very varied. However, all autonomous communities have a person-centred care model in which advance care planning is the framework and there is a national registry that allows citizens to officially record their advance directives with guarantees of confidentiality and accessibility, in addition to freedom and the absence of coercion [19].

Undoubtedly, being aware of the care and treatment preferences of patients can help professionals make decisions that are better adjusted to the wishes of these individuals, such as the transfers of those who wish to die in their own homes assisted by professionals who are experts in end-of-life care [20]. Furthermore, it would be possible to reduce unnecessary clinical actions carried out in the emergency departments and services.

As previously mentioned, despite the fact that some studies have assessed health professionals’ knowledge and attitudes towards AD, they did not analyse this issue jointly and comparatively in hospital emergency departments and emergency medical services. Therefore, the main goal of the present study was to assess the levels and relationship of nursing and medical professionals’ knowledge and attitudes towards AD in hospital emergency departments and emergency medical services, and to determine the correlated variables. The predictive variables of favourable attitudes towards AD were analysed as a secondary goal of the study.

## Methods

### Study design, settings, and subjects

This is an observational, descriptive and cross-sectional study conducted in the Emergency Department of Mollet Hospital (Barcelona), Spain and in an emergency medical service in Catalonia, Spain. The guidelines proposed in ‘Strengthening the reporting of observational studies in epidemiology’ (STROBE) were followed for the preparation of the study.

The participants included in the study were nursing and medical professionals linked to both services and institutions, who had a valid employment contract during the period of the survey, and who wished to participate in the study voluntarily. The professionals excluded were those in the ‘recycling’ period, those who habitually worked with the paediatric population, and graduate students in internships. The instrument used was delivered by the service coordinators to the potential participants in person and/or online through the institutional mail.

The present study was approved by the Clinical Board of Emergency Medical Services (Barcelona) and by the Ethics Committee for Drug Research of the Hospital Clínic Barcelona, with reference number HCB/2020/0158. Authorisation was obtained from the nursing and medical directorates of both institutions. The guidelines of the Declaration of Helsinki relating to ethical principles in clinical research were followed. The authors of the original questionnaire authorised its administration in the present study. With respect to the participants, they received oral and written information concerning the study and the voluntary nature of their participation. To ensure confidentiality, the information collected was registered in a database anonymously, using numbers instead of the names of the participants.

### Sample size calculation

The sample was selected by non-probabilistic convenience sampling of a total of 190 professionals. It was calculated using the equation of proportions, estimating a confidence level of 95% and an expected proportion of losses of 15%. The necessary sample was made up of 128 participants. Finally, 173 nursing and medical professionals, representing a participation rate of 91%, participated in the study and were asked to fill in the questionnaire either on paper or via their computer, mobile phone, and/or tablet.

### Questionnaire development

The data collection was performed from January 2019 to February 2020. The questionnaire “Knowledge and attitudes of health professionals in the process of living will declaration process”, with a reliability ranging from 0.5 to 0.88, and a Kappa pre-retest stability of 0.2 [21], was used to assess the participants’ knowledge and attitudes towards AD. This instrument is made up of forty-one items divided into seven blocks, namely: (a) normative aspects; (b) proposals for conceptual definition; (c) proposals for official documentation; (d) proposals for use; (e) proposals for the registration procedure; (f) proposal for attitudes of health professionals at the time of practical application (APA); and (g) proposals for attitudes of health professionals at the time of practical application in complex scenarios (ACS).

In order to assess the levels of knowledge about planning AD in the sample, it was considered relevant to add the following question: “Do you know the meaning of advance directives planning?”.

Sociodemographic data were also collected using a form attached to the instrument, including sociodemographic and professional variables (sex, age, academic discipline, service, graduate training and/or master's degree in bioethics, years of experience in the service, and work shift). The response format was open-ended, dichotomous, and polychotomous.

### Statistical analysis

The compilation of the responses was performed using the SPSS V.24.0 software for Windows^©^. The statistical analysis of the data was carried out using the R V.4.0.1 software. The normality of continuous variables was determined using QQ plots and the Shapiro-Wilks test. Variables that followed a normal distribution were presented as means (standard deviation). The differences were calculated using the Student's *t*-test for two groups, or ANOVA for more than two groups, correcting for multiple comparisons with Tukey test. Variables that did not follow a normal distribution were presented with the medians (interquartile range). The differences were tested using the nonparametric Mann–Whitney *U* test for two groups, or Kruskal–Wallis for more than two groups, correcting for multiple comparisons using the Benjamin and Hochberg test. Categorical variables were represented with frequency (percentage) and the two groups were compared using the Chi-square test or Fisher's exact test.

A Likert-type measurement scale was created from the APA and ACS categorical variables, assigning their degree of agreement or disagreement on a graduated rating scale from 1 to 5.

Then, ranges of scores were created from the 25th, 50th, and 75th percentiles to determine the APA and ACS of professionals who had favourable, intermediate, or unfavourable attitudes towards AD. The two scores were considered separately.

Multinomial logistic regression models were created to assess the association between variables that were significant in the bivariate analysis, taking the total scores of APA and ACS classification (unfavourable, intermediate, and favourable attitude) as the response variable. All the models were also adjusted for sex, age, education, and work experience. Values of *p* < 0.05 were considered statistically significant.

## Results

The present study assessed 173 participants, of whom 119 were nurses and 54 physicians. The median age was $\tilde{x}$ = 40 years (33.0–47.0); 48 were men and 125 women. Of the total, 57% (*n* = 99) of the participants worked in the emergency department of the hospital, 43% (*n* = 74) in the emergency medical service, 45% (*n* = 78) on the morning shift, and 39% (*n* = 67) had more than fifteen years’ experience. The remaining sociodemographic characteristics are detailed in Table 1.

**Table 1:** Main characteristics of the participants

	[ALL]	Nursing	Medical	No
	*No.* = 173	*No.* = 119	*No.* = 54	
Age	40.0 [33.0; 47.0]	39.0 [32.2; 45.0]	42.0 [36.0; 52.0]	172
Previous Master's or postgraduate degree	8 (4.62%)	6 (5.04%)	2 (3.70%)	173
Years of experience at the service				173
0–5 years	39 (22.5%)	31 (26.1%)	8 (14.8%)	
6–10 years	39 (22.5%)	22 (18.5%)	17 (31.5%)	
11–15 years	28 (16.2%)	20 (16.8%)	8 (14.8%)	
+ 15 years	67 (38.7%)	46 (38.7%)	21 (38.9%)	
Service				173
Hospital emergency department	99 (57.2%)	74 (62.2%)	25 (46.3%)	
Emergency medical service	74 (42.8%)	45 (37.8%)	29 (53.7%)	
Work shift				173
Morning	78 (45.1%)	54 (45.4%)	24 (44.4%)	
Afternoon	32 (18.5%)	24 (20.2%)	8 (14.8%)	
Night	63 (36.4%)	41 (34.5%)	22 (40.7%)	

With respect to knowledge, 53% (*n* = 92) knew that AD declarations could not replace informed consents, 72% (*n* = 124) knew that AD declarations were valid throughout the Spanish territory, 72% (*n* = 125) knew that an admitted individual could make an AD declaration, 57% (*n* = 99) acknowledged that health professionals should provide information about AD to patients, and only 43% (*n* = 74) knew the meaning of AD planning. On the other hand, 74% (*n* = 128) did not know the documentation that should be provided when making an AD declaration, 65% (*n* = 113) did not know where to register AD, 65% (*n* = 112) did not know where to consult them, and only 17% (*n* = 30) knew who could consult them once they had been recorded. In addition, 91% (*n* = 158) acknowledged that they were not sufficiently informed about AD, and 50% (*n* = 87) answered N/A when asked about the legal validity of an AD that was not registered.

**Fig. 1:**
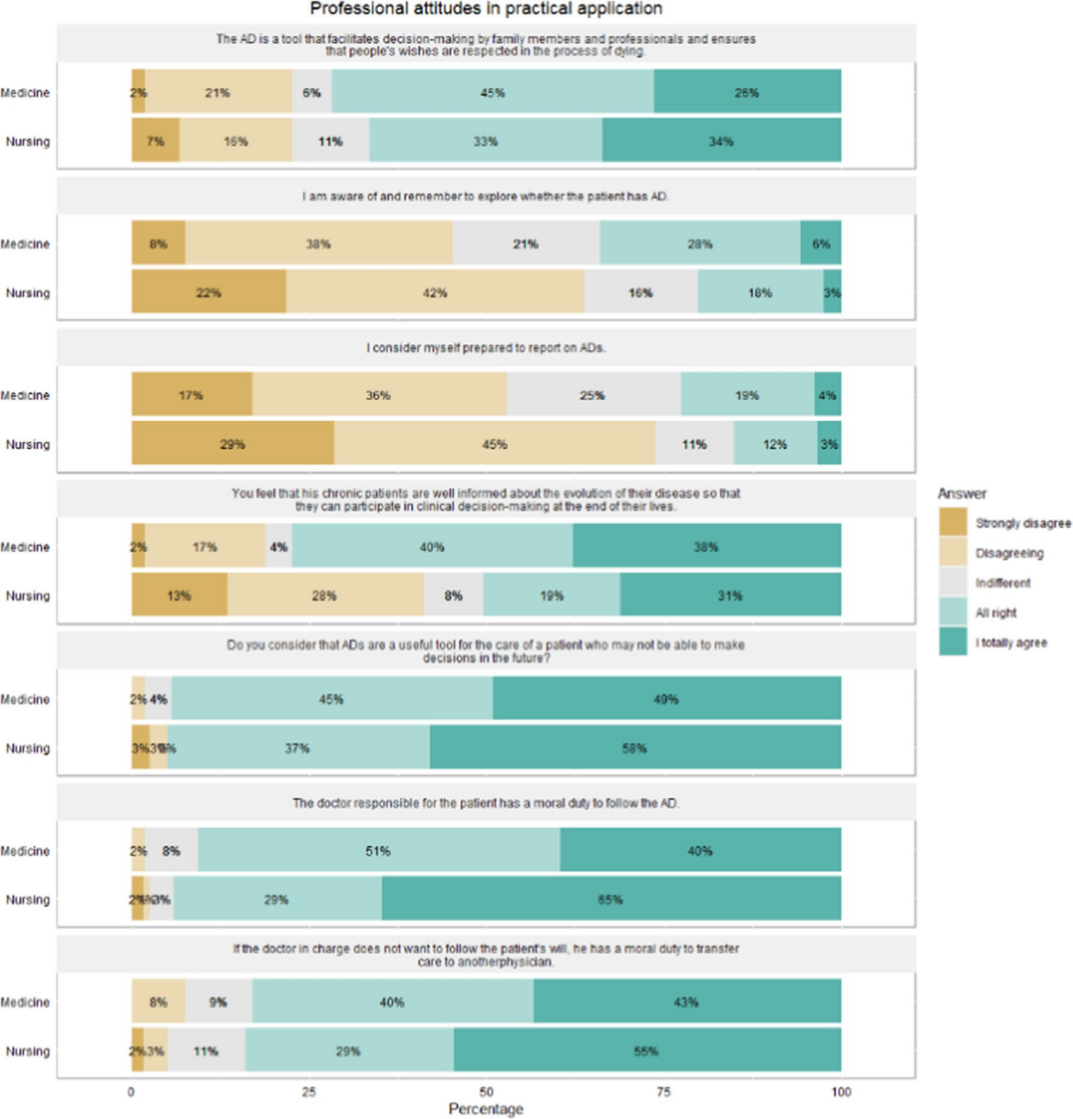
Attitudes of nursing and medical professionals at the time of practical AD application

**Fig. 2:**
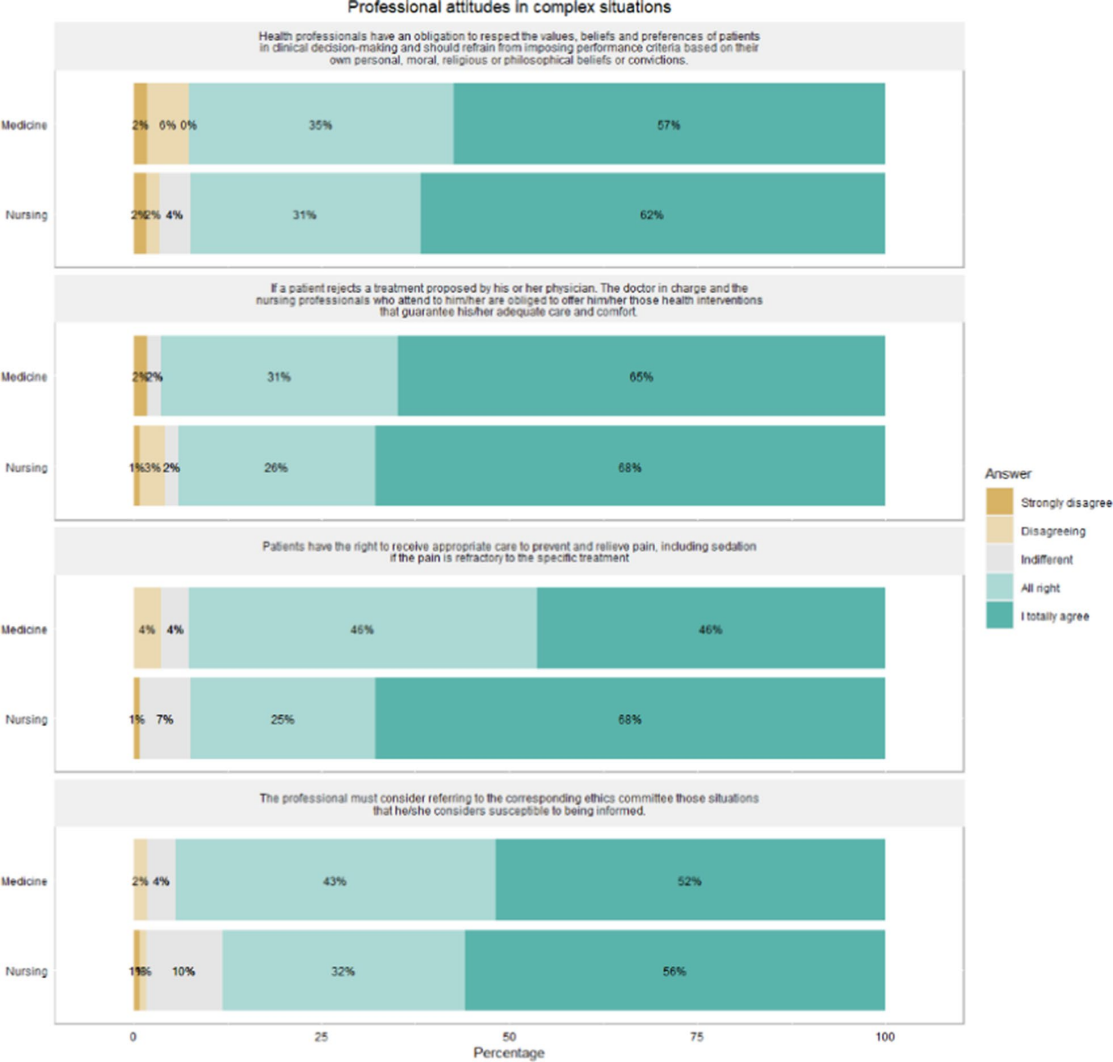
Attitudes of nursing and medical professionals towards AD in complex scenarios

Regarding attitudes, 74% (*n* = 128) of the professionals acknowledged that they had not incorporated consultation of AD in their usual practice (Figs. 1, 2). The comparison of the two groups indicated that nurses (68%; *n* = 80) had a greater tendency to respect the right of the patients to receive appropriate care for prevention and pain relief, including sedation, vs. physicians 46% (*n* = 25) (*p* = 0.005). Nurses also felt that the physician responsible for care had a moral duty to follow the ADs, with 65% (*n* = 77) of nurses strongly agreeing, compared to 40% (*n* = 21) of the doctors (*p* = 0.007). Furthermore, 58% (*n* = 69) of the nursing professionals and 49% (*n* = 26) of the medical professionals considered AD to be very useful as an instrument for healthcare.

The relationship between the level of knowledge and the APA and ACS are detailed in Tables 2 and 3. Looking at the total number of participants, 37% scored favourable on the APA questions, 34% scored intermediate, and the remaining 29% scored unfavourable (Table 2). If we look at the scores for the ACS questions, 43% scored favourable, 23% scored intermediate, and 34% scored unfavourable (Table 3). The best scores in the APA and ACS questions were most frequently observed in those who knew about ADs, the official documentation necessary to perform them, the registration procedures, and their use in clinical practice. This fact was confirmed with a *p* value < 0.05.

**Table 2:** Relationship between the levels of knowledge and attitudes in practical application (APA)

	[ALL]	Unfavourable attitude towards AD	Intermediate attitude towards AD	Favourable attitude towards AD	*p*. overall	*N*
	*N* = 172	*N* = 50 (29*.*1%)	*N* = 58 (33.7%)	*N* = 64 (37.2%)		
Can the declaration of AD replace Informed Consent?					0.028	171
Yes	40 (23.4%)	8 (16.0%)	9 (15.8%)	23 (35.9%)		
No	91 (53.2%)	26 (52.0%)	34 (59.6%)	31 (48.4%)		
N/A	40 (23.4%)	16 (32.0%)	14 (24.6%)	10 (15.6%)		
Is one of the aims of Law 2/2010 (Spain) to ensure the autonomy of patients and respect for their will in the dying process, only when there is a living will?					0.298	171
Yes	37 (21.6%)	12 (24.0%)	9 (15.8%)	16 (25.0%)		
No	70 (40.9%)	17 (34.0%)	23 (40.4%)	30 (46.9%)		
N/A	64 (37.4%)	21 (42.0%)	25 (43.9%)	18 (28.1%)		
Can persons with a judicial resolution of incapacity register a living will if it does not specify this in the resolution?					0.272	170
Yes	40 (23.5%)	10 (20.0%)	14 (25.0%)	16 (25.0%)		
No	55 (32.4%)	13 (26.0%)	16 (28.6%)	26 (40.6%)		
N/A	75 (44.1%)	27 (54.0%)	26 (46.4%)	22 (34.4%)		
Are living wills valid throughout Spain?					0.039	172
Yes	123 (71.5%)	28 (56.0%)	44 (75.9%)	51 (79.7%)		
No	8 (4.65%)	4 (8.00%)	1 (1.72%)	3 (4.69%)		
N/A	41 (23.8%)	18 (36.0%)	13 (22.4%)	10 (15.6%)		
Are health professionals in Catalonia obliged to inform about AD?					0.358	172
Yes	73 (42.4%)	17 (34.0%)	25 (43.1%)	31 (48.4%)		
No	33 (19.2%)	13 (26.0%)	8 (13.8%)	12 (18.8%)		
N/A	66 (38.4%)	20 (40.0%)	25 (43.1%)	21 (32.8%)		
Can the representative appointed in the living will have his or her functions limited by the person granting the Living Will whom he or she represents?					0.423	172
Yes	87 (50.6%)	24 (48.0%)	27 (46.6%)	36 (56.2%)		
No	23 (13.4%)	10 (20.0%)	7 (12.1%)	6 (9.38%)		
N/A	62 (36.0%)	16 (32.0%)	24 (41.4%)	22 (34.4%)		
The living will must always be taken into account, regardless of the patient's state of consciousness					0.492	172
Yes	120 (69.8%)	33 (66.0%)	42 (72.4%)	45 (70.3%)		
No	38 (22.1%)	10 (20.0%)	12 (20.7%)	16 (25.0%)		
N/A	14 (8.14%)	7 (14.0%)	4 (6.90%)	3 (4.69%)		
In the case of deceased persons, does only their legal representative have access to the declaration of a Living Will?					0.809	172
Yes	38 (22.1%)	10 (20.0%)	14 (24.1%)	14 (21.9%)		
No	64 (37.2%)	17 (34.0%)	20 (34.5%)	27 (42.2%)		
N/A	70 (40.7%)	23 (46.0%)	24 (41.4%)	23 (35.9%)		
Do you understand the meaning of limitation of therapeutic effort?					0.140	172
Yes	156 (90.7%)	49 (98.0%)	53 (91.4%)	54 (84.4%)		
No	7 (4.07%)	0 (0.00%)	3 (5.17%)	4 (6.25%)		
N/A	9 (5.23%)	1 (2.00%)	2 (3.45%)	6 (9.38%)		
Have you learned about the meaning and usefulness of the ACP:					0.031	172
Yes	74 (43.0%)	19 (38.0%)	19 (32.8%)	36 (56.2%)		
No	66 (38.4%)	24 (48.0%)	23 (39.7%)	19 (29.7%)		
N/A	32 (18.6%)	7 (14.0%)	16 (27.6%)	9 (14.1%)		
Do you know the documents necessary to carry out an AD					0.292	172
Yes	79 (45.9%)	21 (42.0%)	25 (43.1%)	33 (51.6%)		
No	6 (3.49%)	0 (0.00%)	4 (6.90%)	2 (3.12%)		
N/A	87 (50.6%)	29 (58.0%)	29 (50.0%)	29 (45.3%)		
Registration of the AD can only be made in person by the holder					0.600	172
Yes	66 (38.4%)	18 (36.0%)	20 (34.5%)	28 (43.8%)		
No	50 (29.1%)	16 (32.0%)	15 (25.9%)	19 (29.7%)		
N/A	56 (32.6%)	16 (32.0%)	23 (39.7%)	17 (26.6%)		
At present, ADs containing, exceptionally, elements contrary to the legislation in force are accepted					0.534	172
Yes	16 (9.30%)	3 (6.00%)	4 (6.90%)	9 (14.1%)		
No	50 (29.1%)	16 (32.0%)	15 (25.9%)	19 (29.7%)		
N/A	106 (61.6%)	31 (62.0%)	39 (67.2%)	36 (56.2%)		
Knows who is entitled to access the content of Ads					0.009	172
Yes	36 (20.9%)	7 (14.0%)	8 (13.8%)	21 (32.8%)		
No	76 (44.2%)	26 (52.0%)	22 (37.9%)	28 (43.8%)		
N/A	60 (34.9%)	17 (34.0%)	28 (48.3%)	15 (23.4%)		
In his/her usual practice, has consulted the AD register					0.009	172
Yes	55 (32.0%)	9 (18.0%)	17 (29.3%)	29 (45.3%)		
No	111 (64.5%)	37 (74.0%)	40 (69.0%)	34 (53.1%)		
N/A	6 (3.49%)	4 (8.00%)	1 (1.72%)	1 (1.56%)		
The professional has an obligation to provide information on AD					0.276	172
Yes	99 (57.6%)	23 (46.0%)	34 (58.6%)	42 (65.6%)		
No	13 (7.56%)	4 (8.00%)	4 (6.90%)	5 (7.81%)		
N/A	60 (34.9%)	23 (46.0%)	20 (34.5%)	17 (26.6%)		
AD consultation is part of his or her regular practice					0.002	172
Yes	26 (15.1%)	4 (8.00%)	3 (5.17%)	19 (29.7%)		
No	127 (73.8%)	41 (82.0%)	47 (81.0%)	39 (60.9%)		
N/A	19 (11.0%)	5 (10.0%)	8 (13.8%)	6 (9.38%)		
Health professionals should always consult the AD					0.229	172
Yes	31 (18.0%)	6 (12.0%)	9 (15.5%)	16 (25.0%)		
No	95 (55.2%)	27 (54.0%)	32 (55.2%)	36 (56.2%)		
N/A	46 (26.7%)	17 (34.0%)	17 (29.3%)	12 (18.8%)		
You think you have enough information about AD					0.508	172
Yes	7 (4.07%)	1 (2.00%)	2 (3.45%)	4 (6.25%)		
No	158 (91.9%)	47 (94.0%)	52 (89.7%)	59 (92.2%)		
N/A	7 (4.07%)	2 (4.00%)	4 (6.90%)	1 (1.56%)		
Knows where an AD is consulted					0.052	172
Yes	46 (26.7%)	11 (22.0%)	11 (19.0%)	24 (37.5%)		
No	111 (64.5%)	36 (72.0%)	38 (65.5%)	37 (57.8%)		
N/A	15 (8.72%)	3 (6.00%)	9 (15.5%)	3 (4.69%)		
Knows the documentation to be provided to register an AD					0.001	172
Yes	31 (18.0%)	3 (6.00%)	7 (12.1%)	21 (32.8%)		
No	127 (73.8%)	43 (86.0%)	43 (74.1%)	41 (64.1%)		
N/A	14 (8.14%)	4 (8.00%)	8 (13.8%)	2 (3.12%)		
The AD is legally valid even if it is not registered					0.064	172
Yes	52 (30.2%)	14 (28.0%)	14 (24.1%)	24 (37.5%)		
No	34 (19.8%)	11 (22.0%)	7 (12.1%)	16 (25.0%)		
N/A	86 (50.0%)	25 (50.0%)	37 (63.8%)	24 (37.5%)		
Find out where you have to go to register an AD					0.440	172
Yes	36 (20.9%)	7 (14.0%)	12 (20.7%)	17 (26.6%)		
No	113 (65.7%)	34 (68.0%)	38 (65.5%)	41 (64.1%)		
N/A	23 (13.4%)	9 (18.0%)	8 (13.8%)	6 (9.38%)		
Will only the AD registered in your Autonomous Community be valid?					0.095	172
Yes	22 (12.8%)	5 (10.0%)	5 (8.62%)	12 (18.8%)		
No	50 (29.1%)	14 (28.0%)	13 (22.4%)	23 (35.9%)		
N/A	100 (58.1%)	31 (62.0%)	40 (69.0%)	29 (45.3%)		
Can a person admitted to hospital register his or her AD?					0.168	172
Yes	124 (72.1%)	31 (62.0%)	44 (75.9%)	49 (76.6%)		
No	1 (0.58%)	0 (0.00%)	0 (0.00%)	1 (1.56%)		
N/A	47 (27.3%)	19 (38.0%)	14 (24.1%)	14 (21.9%)		
The appointment to register AD can only be requested through the app *Salud* *Responde* (*Health* *Responds*)					0.033	171
Yes	52 (30.4%)	13 (26.5%)	15 (25.9%)	24 (37.5%)		
No	31 (18.1%)	4 (8.16%)	11 (19.0%)	16 (25.0%)		
N/A	88 (51.5%)	32 (65.3%)	32 (55.2%)	24 (37.5%)		
Is the existence of a representative mandatory for ADs?					0.009	172
Yes	85 (49.4%)	17 (34.0%)	26 (44.8%)	42 (65.6%)		
No	15 (8.72%)	4 (8.00%)	7 (12.1%)	4 (6.25%)		
N/A	72 (41.9%)	29 (58.0%)	25 (43.1%)	18 (28.1%)		
Only physicians are authorised to consult the AD register?					0.474	172
Yes	72 (41.9%)	20 (40.0%)	20 (34.5%)	32 (50.0%)		
No	4 (2.33%)	1 (2.00%)	2 (3.45%)	1 (1.56%)		
N/A	96 (55.8%)	29 (58.0%)	36 (62.1%)	31 (48.4%)		
Are there other groups in addition to physicians who are authorised to consult the register?					0.242	172
Yes	50 (29.1%)	14 (28.0%)	13 (22.4%)	23 (35.9%)		
No	36 (20.9%)	8 (16.0%)	12 (20.7%)	16 (25.0%)		
N/A	86 (50.0%)	28 (56.0%)	33 (56.9%)	25 (39.1%)		
Do you know who can consult the AD once it has been entered in the Register?					0.474	172
Yes	30 (17.4%)	8 (16.0%)	7 (12.1%)	15 (23.4%)		
No	57 (33.1%)	16 (32.0%)	19 (32.8%)	22 (34.4%)		
N/A	85 (49.4%)	26 (52.0%)	32 (55.2%)	27 (42.2%)

**Table 3:** Relationship between the levels of knowledge and attitudes in complex scenarios (ACS)

	[ALL]	Unfavourable attitude towards AD	Intermediate attitude towards AD	Favourable attitude towards AD	*p*. overall	*N*
	*N* = 172	*N* = 59 (34*.*3%)	*N* = 39 (22*.*7%)	*N* = 74 (43%)		
Can the declaration of AD replace Informed Consent?					0.359	171
Yes	40 (23.4%)	12 (20.3%)	9 (23.7%)	19 (25.7%)		
No	91 (53.2%)	28 (47.5%)	23 (60.5%)	40 (54.1%)		
N/A	40 (23.4%)	19 (32.2%)	6 (15.8%)	15 (20.3%)		
Is one of the aims of Law 2/2010 (Spain) to ensure the autonomy of patients and respect for their will in the dying process, only when there is a living will?					0.678	171
Yes	37 (21.6%)	9 (15.3%)	9 (23.7%)	19 (25.7%)		
No	71 (41.5%)	26 (44.1%)	15 (39.5%)	30 (40.5%)		
N/A	63 (36.8%)	24 (40.7%)	14 (36.8%)	25 (33.8%)		
Can persons with a judicial resolution of incapacity register a Living Will if it does not specify this in the resolution?					0.057	170
Yes	40 (23.5%)	13 (22.0%)	8 (21.6%)	19 (25.7%)		
No	56 (32.9%)	15 (25.4%)	9 (24.3%)	32 (43.2%)		
N/A	74 (43.5%)	31 (52.5%)	20 (54.1%)	23 (31.1%)		
Are Living Wills valid throughout Spain?					0.006	172
Yes	124 (72.1%)	35 (59.3%)	26 (66.7%)	63 (85.1%)		
No	8 (4.65%)	3 (5.08%)	2 (5.13%)	3 (4.05%)		
N/A	40 (23.3%)	21 (35.6%)	11 (28.2%)	8 (10.8%)		
Are health professionals in Catalonia obliged to inform about AD?					0.348	172
Yes	72 (41.9%)	20 (33.9%)	16 (41.0%)	36 (48.6%)		
No	33 (19.2%)	12 (20.3%)	10 (25.6%)	11 (14.9%)		
N/A	67 (39.0%)	27 (45.8%)	13 (33.3%)	27 (36.5%)		
Can the representative appointed in the living will have his or her functions limited by the person granting the living will whom he or she represents?					0.710	172
Yes	87 (50.6%)	27 (45.8%)	18 (46.2%)	42 (56.8%)		
No	23 (13.4%)	9 (15.3%)	5 (12.8%)	9 (12.2%)		
N/A	62 (36.0%)	23 (39.0%)	16 (41.0%)	23 (31.1%)		
The living will must always be taken into account, regardless of the patient's state of consciousness					0.099	172
Yes	121 (70.3%)	39 (66.1%)	25 (64.1%)	57 (77.0%)		
No	37 (21.5%)	11 (18.6%)	12 (30.8%)	14 (18.9%)		
N/A	14 (8.14%)	9 (15.3%)	2 (5.13%)	3 (4.05%)		
In the case of deceased persons, does only their legal representative have access to the declaration of a living will?					0.414	172
Yes	37 (21.5%)	12 (20.3%)	11 (28.2%)	14 (18.9%)		
No	64 (37.2%)	20 (33.9%)	11 (28.2%)	33 (44.6%)		
N/A	71 (41.3%)	27 (45.8%)	17 (43.6%)	27 (36.5%)		
Do you understand the meaning of limitation of therapeutic effort?					0.762	172
Yes	156 (90.7%)	55 (93.2%)	36 (92.3%)	65 (87.8%)		
No	7 (4.07%)	1 (1.69%)	1 (2.56%)	5 (6.76%)		
N/A	9 (5.23%)	3 (5.08%)	2 (5.13%)	4 (5.41%)		
Learn about the meaning and usefulness of the ACP					0.487	172
Yes	74 (43.0%)	25 (42.4%)	16 (41.0%)	33 (44.6%)		
No	66 (38.4%)	21 (35.6%)	19 (48.7%)	26 (35.1%)		
N/A	32 (18.6%)	13 (22.0%)	4 (10.3%)	15 (20.3%)		
Knows the documents necessary to carry out an AD					0.367	172
Yes	79 (45.9%)	24 (40.7%)	17 (43.6%)	38 (51.4%)		
No	6 (3.49%)	4 (6.78%)	0 (0.00%)	2 (2.70%)		
N/A	87 (50.6%)	31 (52.5%)	22 (56.4%)	34 (45.9%)		
The registration of the AD can only be made in person by the holder					0.823	172
Yes	66 (38.4%)	24 (40.7%)	13 (33.3%)	29 (39.2%)		
No	49 (28.5%)	16 (27.1%)	14 (35.9%)	19 (25.7%)		
N/A	57 (33.1%)	19 (32.2%)	12 (30.8%)	26 (35.1%)		
At present, ADs containing, exceptionally, elements contrary to the legislation in force are accepted					0.716	172
Yes	16 (9.30%)	5 (8.47%)	3 (7.69%)	8 (10.8%)		
No	51 (29.7%)	17 (28.8%)	15 (38.5%)	19 (25.7%)		
N/A	105 (61.0%)	37 (62.7%)	21 (53.8%)	47 (63.5%)		
Knows who is entitled to access the content of Ads					0.679	172
Yes	36 (20.9%)	9 (15.3%)	10 (25.6%)	17 (23.0%)		
No	77 (44.8%)	28 (47.5%)	18 (46.2%)	31 (41.9%)		
N/A	59 (34.3%)	22 (37.3%)	11 (28.2%)	26 (35.1%)		
In his/her usual practice, he or she has consulted the AD register					0.052	172
Yes	55 (32.0%)	14 (23.7%)	15 (38.5%)	26 (35.1%)		
No	111 (64.5%)	40 (67.8%)	23 (59.0%)	48 (64.9%)		
N/A	6 (3.49%)	5 (8.47%)	1 (2.56%)	0 (0.00%)		
The professional has an obligation to provide information on AD					0.193	172
Yes	98 (57.0%)	27 (45.8%)	26 (66.7%)	45 (60.8%)		
No	14 (8.14%)	8 (13.6%)	2 (5.13%)	4 (5.41%)		
N/A	60 (34.9%)	24 (40.7%)	11 (28.2%)	25 (33.8%)		
AD consultation is part of his or her regular practice					0.369	172
Yes	26 (15.1%)	8 (13.6%)	4 (10.3%)	14 (18.9%)		
No	127 (73.8%)	41 (69.5%)	32 (82.1%)	54 (73.0%)		
N/A	19 (11.0%)	10 (16.9%)	3 (7.69%)	6 (8.11%)		
Health professionals should always consult the AD					0.295	172
Yes	31 (18.0%)	11 (18.6%)	9 (23.1%)	11 (14.9%)		
No	94 (54.7%)	28 (47.5%)	24 (61.5%)	42 (56.8%)		
N/A	47 (27.3%)	20 (33.9%)	6 (15.4%)	21 (28.4%)		
You think you have enough information about AD					0.430	172
Yes	7 (4.07%)	2 (3.39%)	2 (5.13%)	3 (4.05%)		
No	157 (91.3%)	52 (88.1%)	37 (94.9%)	68 (91.9%)		
N/A	8 (4.65%)	5 (8.47%)	0 (0.00%)	3 (4.05%)		
Knows where an AD is consulted					0.680	172
Yes	46 (26.7%)	13 (22.0%)	13 (33.3%)	20 (27.0%)		
No	111 (64.5%)	39 (66.1%)	24 (61.5%)	48 (64.9%)		
N/A	15 (8.72%)	7 (11.9%)	2 (5.13%)	6 (8.11%)		
Knows the documentation to be provided to register an AD					0.065	172
Yes	31 (18.0%)	6 (10.2%)	6 (15.4%)	19 (25.7%)		
No	127 (73.8%)	45 (76.3%)	32 (82.1%)	50 (67.6%)		
N/A	14 (8.14%)	8 (13.6%)	1 (2.56%)	5 (6.76%)		
The AD is legally valid even if it is not registered					0.557	172
Yes	52 (30.2%)	17 (28.8%)	10 (25.6%)	25 (33.8%)		
No	34 (19.8%)	15 (25.4%)	6 (15.4%)	13 (17.6%)		
N/A	86 (50.0%)	27 (45.8%)	23 (59.0%)	36 (48.6%)		
Find out where you have to go to register an AD					0.061	172
Yes	36 (20.9%)	8 (13.6%)	11 (28.2%)	17 (23.0%)		
No	112 (65.1%)	37 (62.7%)	25 (64.1%)	50 (67.6%)		
N/A	24 (14.0%)	14 (23.7%)	3 (7.69%)	7 (9.46%)		
Will only the AD registered in your Autonomous Community be valid?					0.951	172
Yes	22 (12.8%)	7 (11.9%)	5 (12.8%)	10 (13.5%)		
No	51 (29.7%)	16 (27.1%)	11 (28.2%)	24 (32.4%)		
N/A	99 (57.6%)	36 (61.0%)	23 (59.0%)	40 (54.1%)		
Can a person admitted to hospital register his or her ADs?					0.001	172
Yes	124 (72.1%)	35 (59.3%)	25 (64.1%)	64 (86.5%)		
No	1 (0.58%)	1 (1.69%)	0 (0.00%)	0 (0.00%)		
N/A	47 (27.3%)	23 (39.0%)	14 (35.9%)	10 (13.5%)		
The appointment to register AD can only be requested through the app *Salud* *Responde* (*Health* *Responds*)					0.011	171
Yes	52 (30.4%)	14 (24.1%)	16 (41.0%)	22 (29.7%)		
No	31 (18.1%)	7 (12.1%)	3 (7.69%)	21 (28.4%)		
N/A	88 (51.5%)	37 (63.8%)	20 (51.3%)	31 (41.9%)		
Is the existence of a representative mandatory for ADs?					0.480	172
Yes	85 (49.4%)	25 (42.4%)	20 (51.3%)	40 (54.1%)		
No	15 (8.72%)	4 (6.78%)	3 (7.69%)	8 (10.8%)		
N/A	72 (41.9%)	30 (50.8%)	16 (41.0%)	26 (35.1%)		
Only physicians are authorised to consult the AD register?					0.374	172
Yes	72 (41.9%)	21 (35.6%)	15 (38.5%)	36 (48.6%)		
No	4 (2.33%)	1 (1.69%)	2 (5.13%)	1 (1.35%)		
N/A	96 (55.8%)	37 (62.7%)	22 (56.4%)	37 (50.0%)		
Are there other groups in addition to physicians who are authorised to consult the register?					0.115	172
Yes	49 (28.5%)	13 (22.0%)	16 (41.0%)	20 (27.0%)		
No	36 (20.9%)	12 (20.3%)	4 (10.3%)	20 (27.0%)		
N/A	87 (50.6%)	34 (57.6%)	19 (48.7%)	34 (45.9%)		
Do you know who can consult the AD once it has been entered in the Register?					0.161	172
Yes	29 (16.9%)	5 (8.47%)	10 (25.6%)	14 (18.9%)		
No	57 (33.1%)	19 (32.2%)	11 (28.2%)	27 (36.5%)		
N/A	86 (50.0%)	35 (59.3%)	18 (46.2%)	33 (44.6%)		

The multivariable analysis indicated a statistically significant relationship between the variables of service and exhibiting greater predisposition on the part of the professionals to consult the AD of the patients. It was observed that the fact of belonging to the emergency medical service was a predictor variable of a favourable attitude (OR 2.49 [95% CI 1.06–5.88]; *p* = 0.037) *vs.* an unfavourable one, taking those of the emergency department of the hospital as reference. Intermediate *vs.* unfavourable attitudes were not statistically significant (OR 1.5 [95% CI 0.63–3.59]; *p* = 0.357). In addition, it was found that with increasing age and years of experience of the professionals there was not a greater probability of obtaining a favourable attitude in APA (Table 4).

**Table 4:** Sociodemographic factors that affect attitudes towards AD in its practical application (APA)

	Intermediate attitude towards AD OR (95% CI)	*p* value	Favourable attitude towards AD OR (95% CI)	*p* value
Intercept	0.15 (0.01; 2.09)	0.158	0.08 (0.01; 1.21)	0.069
Sex: Woman	1.09 (0.43; 2.81)	0.852	1.47 (0.56; 3.81)	0.433
Age	1.06 (0.98; 1.14)	0.151	1.05 (0.97; 1.13)	0.238
Discipline: Medicine	0.86 (0.33; 2.25)	0.759	1.34 (0.52; 3.42)	0.547
Years of experience: 6–10	0.6 (0.19; 1.85)	0.371	0.95 (0.29; 3.14)	0.932
Years of experience: 11–15	0.77 (0.19; 3.13)	0.715	0.93 (0.21; 4.19)	0.928
Years of experience: + 15	0.73 (0.13; 4.02)	0.713	1.74 (0.3; 10)	0.535
Service: Emergency medical service	1.5 (0.63; 3.59)	0.357	2.49 (1.06; 5.88)	0.037

*AD* advance directives, *CI* confidence interval

Regarding attitudes of health professionals at the time of practical application in complex scenarios (ACS), it was observed that belonging to the emergency medical service meant a greater likelihood of obtaining a favourable attitude (OR 3.65 [95% CI 1.58 − 8.41]; *p* = 0.002) or intermediate as opposed to an unfavourable one, in comparison to those of the hospital emergency department. Working the afternoon shift (OR 3.3 [95% CI 1.1 − 9.84]; *p* = 0.032) and/or night shift (OR 2[95% CI 0.86–4.68]; *p* = 0.11) was also a predictor variable for obtaining a higher score in ACS, in comparison to working the morning shift (Table 5).

**Table 5:** Sociodemographic factors that affect attitudes towards AD in complex scenarios (ACS)

	Intermediate attitude towards AD OR (95% CI)	*p* value	Favourable attitude towards AD OR (95% CI)	*p* value
Intercept	0.31 (0.02; 5.3)	0.417	0.11 (0.01; 1.43)	0.092
Sex: Woman	1.2 (0.44; 3.3)	0.726	1.34 (0.55; 3.26)	0.516
Age	1.02 (0.94; 1.1)	0.716	1.04 (0.97; 1.12)	0.272
Discipline: Medicine	0.93 (0.35; 2.48)	0.883	0.57 (0.24; 1.37)	0.208
Years of experience: 6–10	0.5 (0.14; 1.72)	0.272	0.63 (0.2; 2.01)	0.433
Years of experience: 11–15	1.36 (0.27; 6.94)	0.71	1.46 (0.32; 6.68)	0.622
Years of experience: + 15	0.48 (0.07; 3.05)	0.436	0.69 (0.13; 3.55)	0.659
Service: Emergency medical service	1.58 (0.61; 4.09)	0.341	3.65 (1.58; 8.41)	0.002
Afternoon shift	1.78 (0.51; 6.16)	0.364	3.3 (1.1; 9.84)	0.032
Night shift	1.88 (0.73; 4.85)	0.193	2 (0.86; 4.68)	0.11

*AD* advance directives, *CI* confidence interval

## Discussion

The results show that there is still insufficient specific knowledge and skills relating to the management of AD in the context of emergency services, in line with previous studies [22–25]. Nevertheless, the present study provides novel aspects regarding which variables are predictors of favourable attitudes towards AD.

In the same line as the results found by Mateos et al. [26] and Marco et al. [25, 27], there was good predisposition of nursing and medical professionals to respect the autonomy of decision making of the patients they served and they even considered ADs as a fundamental tool for making decisions in clinical practice. However, as pointed out by Pérez [28], there was still a lack of knowledge about ADs, their registering mechanisms at a practical level, and the normative aspects that regulated them. According to a phenomenological-hermeneutic study that interviewed 24 emergency care services professionals [13], the lack of knowledge of professionals can act as a barrier and make it difficult to routinely consult ADs in the emergency services. This further emphasises the need to make efforts to raise awareness among emergency professionals of the importance of knowing and consulting patients' ADs as a central element in making decisions in accordance with the wishes and preferences of individuals and as a fundamental tool to mitigate potential ethical and legal conflicts arising from the practice of care in these situations.

A noteworthy finding of the present study is the association between the level of knowledge that nursing and medical professionals have regarding AD and the scores obtained on the APA and ACS scales. This result indicates that the more knowledgeable professionals are about AD, the more inclined they are to consult the AD of patients and respect their wishes and preferences regarding care and/or treatment and, therefore, apply them as a routine consultation in these departments. A study conducted in Germany with emergency physicians revealed that the therapeutic decisions of the professionals were influenced by the existence of ADs in 77% of the cases [29]. In this way, professionals’ consultation of patient ADs could be determined by the knowledge they have about AD. However, the lack of knowledge could represent greater variability of response to the same scenario at the clinical level, especially in a vital risk situation that entails urgent action [26–28]. This variability in the decision-making process could represent a risk of patient rights’ protection, especially with regard to their autonomy in choosing the treatments or interventions according to their preferences [29–31].

On the other hand, it should be noted that no relationship was observed between the age and experience of the professionals with attitudes at the time of practical application (APA) or at in complex scenarios (ACS). These results are in disagreement with other reports, which indicated that older professionals and those with greater experience in the service being the ones who were more compliant with the ADs of the patients they attended [28, 32].

There are two important findings that have not been presented in previous studies and which are directly related to the attitudes of professionals towards ADs in their daily practice and in complex situations. Firstly, the fact that the professionals who worked in the emergency service had more favourable attitudes towards patient ADs than those professionals of the emergency department is a new piece of information. Perhaps the fact of caring for patients in critical situations or at the end of life is a key element to explain the interest or a favourable attitude towards AD.

Secondly, the nurses and physicians on the evening and night shifts of the emergency medical services and the emergency departments were the ones most willing to respect the ADs of the persons attended in a complex situation involving a vital risk to the person, as would be the case of cardio-respiratory arrest. This result may be explained by the lack of time and the heavy workloads to which the professionals working the morning shift are subjected, which may be a clear obstacle to respecting the wishes and preferences of the person in a situation of incapacity to make decisions [13, 22, 28, 32]. This could also be explained by the fact that in the evening and night shifts there are fewer professionals to consult, so that professionals working in these shifts may find the ADs helpful to guide decision-making in difficult situations.

In this line, further studies should examine this finding more deeply and with larger samples in order to determine which factors make professionals of emergency medical services more likely to consult and respect patient ADs and which ones hinder morning shift professionals in respecting people's wishes and preferences.

Finally, the results show that those working the evening and night shift, and those who worked in the emergency medical services, were more likely to consult and respect patient ADs. These variables must be taken into account when designing strategies to improve AD management in intra-hospital and extra-hospital emergency services. Undoubtedly, this can contribute to improving the care and clinical attention of patients and to respect for patient autonomy.

### Limitations

Among the limitations of the present study we should note that the sample size was moderate, although it exceeded those of other similar studies [26, 29]. Also, it is worth mentioning the heterogeneous representativeness of the two groups, since the highest response rate was obtained in nursing. However, the proportion of the two professional groups was certainly not the same in the clinical setting. For this reason, only the trend followed by the professionals' responses was shown in the presentation of the results by professional category. The results by sex were not compared either, since the highest participation in the study was that of women, an issue that should be taken into account if an analysis of the results is to be carried out in terms of sex. Finally, it is worth mentioning that we obtained a minimal proportion of losses for some variables in the questionnaire, and this may have influenced the interpretation of the results.

### Implications for clinical practice

Systematic consultation of patients' ADs in emergency departments and emergency medical services would, firstly, make it possible to adjust treatments and interventions to patients' wishes and preferences, thus favouring person-centred care. Secondly, it would improve decision-making by professionals in complex and rapid response situations and, thirdly, it would reduce conflict situations with patients and relatives that arise from not knowing the advance directives.

## Conclusion

These results highlight the need to redefine and identify new frameworks of action to help implement routine consultation of advance directives in emergency departments and emergency medical services. To this end, it is necessary firstly to train and sensitize nurses and physicians in the emergency departments with new training programs that promote the acquisition of knowledge on the management of advance directives in a practical and experiential way. Secondly, it is crucial that both organisations and institutions become aware of the importance of improving AD consultation processes in order to increase the quality of care, reduce conflicts in these services, and respond appropriately to patients' needs and expectations.

## Data Availability

The data used and/or analyzed during the current study are available from the corresponding author on reasonable request.

## Abbreviations

AD: Advance directives
APA: Attitudes of health professionals at the time of practical application
ACS: Attitudes of health professionals at the time of practical application in complex scenarios
